# In vitro investigation of the effects of exogenous sugammadex on coagulation in orthopedic surgical patients

**DOI:** 10.1186/s12871-018-0519-3

**Published:** 2018-05-24

**Authors:** Il Ok Lee, Young Sung Kim, Hae Wone Chang, Heezoo Kim, Byung Gun Lim, Mido Lee

**Affiliations:** Department of Anesthesiology and Pain Medicine, Korea University Guro Hospital, Korea University College of Medicine, Seoul, Republic of Korea

**Keywords:** Blood coagulation, Sugammadex, Thromboelastography

## Abstract

**Background:**

Previous studies have shown that sugammadex resulted in the prolongation of prothrombin time and activated partial thromboplastin time. In this study, we aimed to investigate the in vitro effects of exogenous sugammadex on the coagulation variables of whole blood in healthy patients who underwent orthopedic surgery.

**Methods:**

The effects of sugammadex on coagulations were assessed using thromboelastography (TEG) in kaolin-activated citrated blood samples taken from 14 healthy patients who underwent orthopedic surgery. The in vitro effects of three different concentrations of sugammadex (42, 193, and 301 μg mL^− 1^) on the TEG profiles were compared with those of the control (0 μg mL^− 1^). Previous studies indicated that these exogenous concentrations correspond to the approximate maximum plasma concentrations achieved after the administration of 4, 16, and 32 mg kg^− 1^ sugammadex to healthy subjects.

**Results:**

Increased sugammadex concentrations were significantly associated with reduced coagulation, as evidenced by increases in reaction time (r), coagulation time, and time to maximum rate of thrombus generation (TMRTG), and decreases in the angle, maximum amplitude, and maximum rate of thrombus generation. Compared with the control, the median percentage change (interquartile range) in the TEG values of the samples treated with the highest exogenous sugammadex concentration was the greatest for r, 53% (26, 67.3%), and TMRTG, 48% (26, 59%).

**Conclusions:**

This in vitro study suggests that supratherapeutic doses of exogenous sugammadex might be associated with moderate hypocoagulation in the whole blood of healthy subjects.

**Trial registration:**

identifier: UMIN000029081, registered 11 September 2017.

## Background

Sugammadex (Bridion®, Merck Sharp & Dohme B.V., Haarlem, The Netherlands), a modified gamma-cyclodextrin, forms a stable complex with steroidal neuromuscular blockers via internal lipophilic bonds. The resultant hydrophilic encapsulated compound does not bind to proteins or the plasma membrane and is eliminated unchanged in the urine [1]. De Kalm and co-workers reported that sugammadex caused a moderate (11–22%) increase in the prothrombin time (PT) and activated partial thromboplastin time (aPTT) in healthy volunteers after doses of 16 and 4 mg kg^− 1^ [2, 3]. In vitro experiments suggest that sugammadex may inhibit the effects of factor Xa on prothrombin-to-thrombin conversion in the common pathway of coagulation and may inhibit factor Xa formation via the intrinsic pathway [2].

Thromboelastography (TEG) provides a real-time viscoelastic profile of patients, from the initial thrombin activation to fibrinolysis, and yields information not available through conventional coagulation studies. TEG is also used as a tool to assess the anticoagulation action of oral anticoagulant drugs [4] and to assist in clinical decisions for patients taking oral anticoagulants requiring urgent surgery [5, 6]. The evaluation of the potential effects of drugs on coagulation variables is important because a combination of more than two drugs that affect coagulation may substantially increase a patient’s risk of bleeding [7].

In this study, we aimed to use TEG to assess the effects of exogenous sugammadex on the coagulation variables of whole blood taken from healthy patients undergoing elective orthopedic surgery.

## Methods

This study was approved by the institutional review board at Korea University Guro Hospital, Seoul, Korea, in 2017 (protocol: KUGH17171-001) and registered with UMIN Clinical Trials Registry (identifier: UMIN000029081). After approval from the ethics committee and obtaining informed consent from the patients, we enrolled 15 healthy subjects, aged between 19 and 45 years, with American Society of Anesthesiologists (ASA) physical status score of 1 or 2, who were undergoing elective orthopedic surgery. Patients who had received aspirin or oral anticoagulants within 14 days of the study, who showed abnormal coagulation laboratory values (platelet count < 100,000, PT-international normalised ratio (PT-INR) > 1.5, or aPTT > 50 s), pregnant women, patients with liver or renal disease, patients with other surgical history within 30 days of the study, and patients taking oral contraceptives were excluded. Blood samples were collected prior to anesthetic induction. An 18-gauge peripheral IV cannula was inserted into the antecubital vein and an initial blood sample was obtained for TEG analysis under minimal stasis with a tourniquet by using the two-syringe technique. The first 4 mL of blood was discarded to avoid tissue contamination. The next 5.4 mL of blood was collected using a 10 mL syringe, and the blood was transferred into two 2.7 mL tubes, each containing 0.5 mL of 0.109 M buffered sodium citrate (Vacutainer; Becton, Dickinson and Company®, Franklin Lakes, NJ, USA). The collected blood samples were immediately mixed gently 5–6 times, incubated for approximately 15–20 min, and then analyzed. Briefly, 1 mL of citrated blood was added into a vial containing kaolin. Then, 52 μL of exogenous sugammadex was mixed with 948 μL of 0.9% saline to yield a 2.4 mM solution of sugammadex. For each patient, one solution of citrated whole blood and three solutions of citrated whole blood mixed with different concentrations of exogenous sugammadex were prepared in separate kaolin vials using micropipettes, as follows:

Control: 1 mL of citrated whole blood or sugammadex solutions at each of the three concentrations.

Citrated whole blood (1 mL) with 42 μg of sugammadex: final exogenous sugammadex concentration = 42 μg mL^− 1^.

Citrated whole blood (1 mL) with 199 μg of sugammadex: final exogenous sugammadex concentration = 193 μg mL^− 1^.

Citrated whole blood (1 mL) with 319 μg of sugammadex: final exogenous sugammadex concentration = 301 μg mL^− 1^.

We selected the three concentrations of exogenous sugammadex as those that corresponded to the approximate maximum mean plasma concentrations achieved after the administration of 4, 16, and 32 mg kg^− 1^ sugammadex to healthy patients in a previous study [7, 8]. Doses of 4 and 16 mg kg^− 1^ are used for the reversal of deep neuromuscular block (when recovery has reached at least 1–2 post-tetanic counts) and the immediate reversal after the administration of rocuronium 1.2 mg kg^− 1^ within 3 min, respectively, and 32 mg kg^− 1^ is a supratherapeutic safe dose for awake patients after the use of rocuronium 1.2 mg kg^− 1^ [9]. At the highest concentration, the volume of sugammadex solution was less than 6% of the whole blood volume.

After mixing 8–10 times by inversion, 340 μL of kaolin-activated blood from each solution was pipetted into a plastic cup in a TEG® 5000 hemostasis analyser pre-warmed to 37 °C (Haemonetics®, Braintree, USA). Each sample was recalcified in a plastic cup through the addition of 20 μL 0.2 M CaCl_2_, and a TEG analysis was performed within 1 min after the samples were reconstituted. Additional venous blood was also obtained to assess baseline values for hematologic and coagulation data (Table 1). PT and aPTT were measured using the STAR Evolution (Diagnostica stago, Parsippany, New Jersey). An abnormal aPTT was defined as a clotting time outside our laboratory’s normal range (30.2-42.4 s). Prolonged PT was defined as a clot time greater than 14.4 s (normal range: 11.3-14.4 s).

**Table 1:** Patient characteristics and a summary of hematological and coagulation variables. The data are presented as the mean (SD) or number of patients

	(*n* = 14)
Age (y)	32 (9)
Sex (M/F)	11/3
Body weight (kg)	83.1 (18.1)
Height (cm)	171.5 (8.2)
HB^a^ (g.dL^−1^)	15.0 (1.2)
Hct^b^ (%)	44.2 (3.6)
Cr^c^ (mg.dL^−1^)	0.8 (0.2)
PT^d^ (%)	106.7 (11.2)
PTINR^e^	0.97 (0.06)
aPTT^f^ (s)	35.2 (4.8)
Platelet (10^3.^μL^−1^)	256 (66.7)

^a^HB, hemoglobin; ^b^Hct, hematocrit; ^c^Cr, creatinine; ^d^PT, prothrombin time; ^e^PT INR, prothrombin time internalization normalization ratio; ^f^aPTT, activated prothrombin time

The variables recorded included the reaction time (r, min), which represented the rate of clot initiation, the coagulation time (k, min), and the angle (α, degrees), which represented the rate of fibrin polymerization. The maximum amplitude (MA, mm) represented the maximum clot strength and was depicted as the greatest width of the TEG tracing. The shear elastic modulus strength (G, dynes cm^− 2^) is a parametric measure of clot firmness expressed in metric units and calculated from the MA according to the following equation: G = (5000 × MA) / (100 – MA). Lysis 30 (Ly30) is an index of fibrinolytic activity.

In addition to conventional TEG parameters, we measured clot kinetic parameters using a graph produced by TEG. These parameters included the maximum rate of thrombus generation (MRTG, dynes cm^− 2^ s), which is the first derivative of the velocity of the increase in clot strength, which starts from the increase in G and ends at the stabilization of the clot strength. In addition, we determined the time to maximum clot strength (TMRTG, min), which is the time necessary to reach the MRTG and reflects the enzymatic contribution to clot formation. Total thrombin generation (TTG), which is the total positive area under the velocity curve, represents the total change in elastic resistance until clot strength stabilization, and depicts thrombin generation.

The TEG was determined by an observer blinded to the group assignment and sampling time. The primary outcomes were the TEG parameters for citrated whole blood and citrated whole blood mixed with exogenous sugammadex, while the secondary outcome was the association between the sugammadex concentration and changes in TEG parameters.

We assessed the r values of the plain whole citrated blood and whole citrated blood containing 42 μg mL^− 1^ sugammadex in an initial pilot sample of five patients undergoing orthopedic surgery. The mean r values were 5.3 ± 0.9 and 6 ± 1.2. Student’s paired *t*-test revealed that the coefficient of correlation was 0.94 with a power of 1.49; therefore, to reach a power of 95% and an alpha level of 0.01, the estimated sample size was 11. We enrolled 15 patients to avoid any possible technical difficulties associated with blood sampling or TEG analysis. The data were assessed for normality by using normality plots and the Kolmogorov-Smirnov test. Changes in individual TEG parameters were assessed by a repeated-measures analysis of variance with Holm-Sidak’s multiple comparison tests. We also performed a mixed model regression analysis for each TEG parameter to evaluate the association between the concentration of exogenous sugammadex and thromboelastographic parameters. The percentage change was calculated for each TEG parameter for all groups. Statistical analyses were conducted using GraphPad Prism version 7.03 for Windows (GraphPad Inc., La Jolla, USA) and Predictive Analytics Software (PASW) statistics 18.0 (SPSS Inc., Chicago, IL, USA). Values of *P* < 0.05 were considered statistically significant.

## Results

Blood samples were obtained from 15 patients with ASA physical status 1–2 who underwent orthopedic surgery. One sample was excluded owing to a technical error in blood sampling that may have affected the values of the TEG parameters. Therefore, the results were analyzed for 14 patients. The demographic and baseline laboratory hematological data of all patients are presented in Table 1.

The TEG parameters and the dynamic parameters are shown in Table 2. The values for the r time, k time, α angle, MA, G, MRTG, and TMRTG in the control group were within the normal range. Only TTG results were above the normal values. There was a significant increase in r time and TMRTG at all tested concentrations of sugammadex and the values were within or above the normal range. There were statistically significant differences in all TEG parameters between the control and the 301 μg mL^− 1^ concentration of sugammadex. There were no statistically significant differences between the 301 and 193 μg mL^− 1^ concentrations of sugammadex.

**Table 2:** Thromboelastographic parameters from citrated whole blood and citrated whole blood treated with exogenous sugammadex (42, 193, and 301 μg mL^− 1^) collected from patients who underwent orthopedic surgery

	Exogenous sugammadex concentration (μg mL^− 1^)				
	0	42	193	301	P
R^a^ (min) 2–8	5.6 (1.2)	6.3 (1.5)***	7.9 (1.9)^◇◇◇,^****	8.2 (2.4)^,^****	< 0.0001
K^b^ (min) 1–3	1.7 (0.4)	1.8 (0.5)	2.3 (0.7)^◇,*^	2.5 (1.2)^□, *,^	0.0018
Angle^c^ (degrees) 55–78	64.5 (8.6)	64.8 (6.7)	54.0 (10.9) ^◇,^*	56.5 (10.8)^□,^*	0.0004
MA^d^ (min) 51–69	67.9 (6.8)	64.8 (6.0)*	61.8 (8.0)*	60.5 (8.9)^□, **^	0.0008
G^e^ (dynes (cm^2^)^−1^) 4.6–10.9 K	11.3 (4.0)	9.6 (2.5)	8.7 (2.9)	8.3 (3.1)*	0.0065
Ly30 (%) 0–8	1.1 (1.0)	1.3 (1.0)	0.9 (1.1)	0.3 (0.5)^□□, *^	0.0461
MRTG^g^ (mm min^−1^) 5–17	12.7 (3.3)	11.8 (3.0)	10.3 (3.4)*	9.9 (3.7)**	0.0021
TMRTG^h^ (min) 6–12	7.0 (1.5)	7.7 (1.7)**	9.5 (2.3)^◇◇◇,^***	10.0 (2.9)^□□□,^***	< 0.0001
TTG^i^ (mm min^−1^) 584–796	821 (83.1)	781.2 (70.3)*	749.7 (97.0)*	733.7 (106.2)**	0.0010

The values presented are the mean (SD), *n* = 14. Holm-Sidak’s multiple comparisons test was performed to compare the significance of the differences between each study solution. ^a^R, reaction time to clot formation; ^b^K, time to achieve a clot strength of 20 mm amplitude; ^c^Angle, rate of clot growth; ^d^MA, maximum amplitude of clot strength; ^e^G, shear elastic modulus strength; ^g^MRTG, maximum rate of thrombus generation; ^h^TMRTG, time to maximum rate of thrombus generation; ^i^TTG, total thrombin time; Statistically significant differences between; (301 vs 193), □ (301 vs 42), ◇ (193 vs 42) and * (control). Single symbol and * indicate *P* < 0.05 compared with control value; two symbols □□ and ** indicate *P* < 0.01; and three symbols □□□, ◇◇◇, and *** indicate *P* < 0.001, **** indicate *P* < 0.0001

**Fig. 1:**
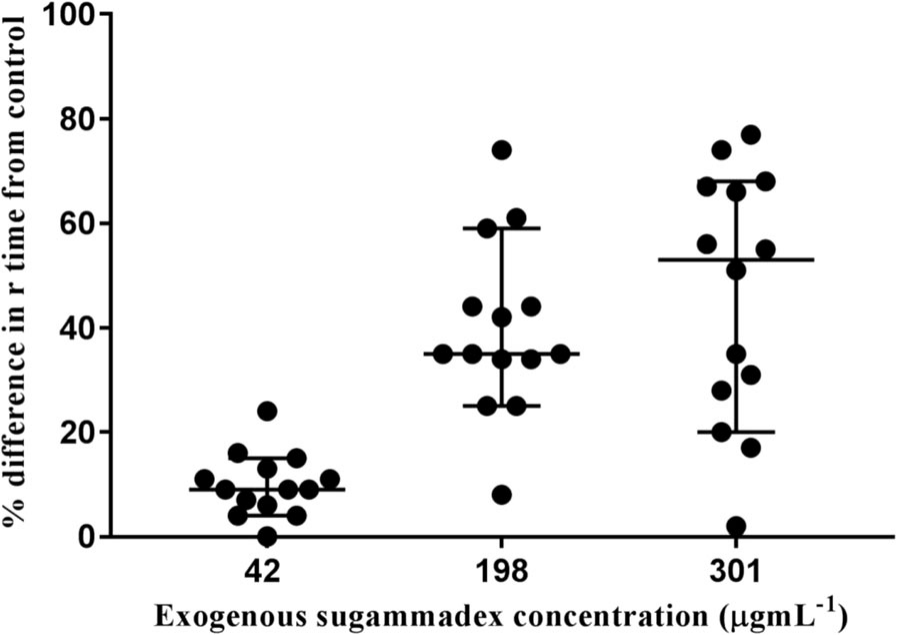
The relationship between the r time % difference from control and the concentration of exogenous sugammadex

The average median (interquartile range) and mean (SD) percentage difference between the control and the highest concentration of sugammadex were as follows: r time = 53% (26, 67.3%), k time = 39.5% (18.8, 61%), α angle = − 11.7% (16.9%), MA = − 10.5% (− 21, − 2.5%), MRTG = − 22.6% (20.7%), TMRTG = 48% (26, 59%), and TTG = − 9.5% (− 20.3, − 2.5%). Figure 1 shows the relationship between the % difference in r time from the control and sugammadex concentrations across the volunteers.

The mixed model regression analysis indicated that an increase in sugammadex concentration was significantly associated with increases in r time, k time, and TMRTG, and with decreases in α angle, MA, G, Ly30, MRTG, and TTG (*P* < 0.05, respectively) (Table 3).

**Table 3:** Linear mixed-effects regression model for the effect of exogenous sugammadex on thromboelastographic parameters

	Intercept	β coefficient (standard error)	*P* value
R^a^ (min) 2–8	5.9	0.008 (0.002)	0.002
K^b^ (min) 1–3	1.7	0.003 (0.001)	0.018
Angle^c^ (degrees) 55–78	64.5	−0.032 (0.011)	0.009
MA^d^ (min) 51–69	66.7	−0.021 (0.009)	0.028
G^e^ (dynes (cm^2^)^−1^) 4.6–10.9 K	10.6	−0.008 (0.003)	0.024
Ly30 (%) 0–8	1.3	−0.003 (0.001)	0.010
MRTG^g^ (mm min^−1^) 5–17	12.3	−0.009 (0.004)	0.036
TMRTG^h^ (min) 6–12	7.2	0.010 (0.003)	0.002
TTG^i^ (mm min^−1^) 584–796	805.8	−0.246 (0.108)	0.033

The average baseline r time calculated using mixed model repeated linear regression was 5.9 min; the rate of change of r time was 0.008 min per 1 μg mL^− 1^ increase in exogenous sugammadex concentration. ^a^R, reaction time to clot formation; ^b^K, time to achieve a clot strength of 20 mm amplitude; ^c^Angle, rate of clot growth; ^d^MA, maximum amplitude of clot strength; ^e^G, shear elastic modulus strength; ^g^MRTG, maximum rate of thrombus generation; ^h^TMRTG, time to maximum rate of thrombus generation; ^i^TTG, total thrombin time

## Discussion

This study was conducted to investigate the effects of exogenous sugammadex on TEG parameters. The two highest concentrations of sugammadex (198 and 301 μg mL^− 1^) significantly altered all TEG parameters. However, at the therapeutic concentration (42 μg mL^− 1^), sugammadex did not induce clinically meaningful changes in any parameters. The r time, α angle, and TMRTG level were significantly different between the lowest and the two highest concentrations of sugammadex, which was indicative of a dose-dependent response. Previous investigations have shown that the anticoagulation effects of sugammadex measured by conventional coagulation tests occurred at similar magnitudes in in vivo and in vitro studies, with dose-dependent effects observed [2]. The highest concentration of sugammadex increased the r time by more than 50% compared with the control. In this study, we found direct associations between the estimated concentration of sugammadex in vitro and all individual TEG parameters by using previously reported mixed-effects modelling methods [10]. The changes in all parameters indicated modest anticoagulant action. The observation of reduced fibrinolysis in the two higher concentrations of sugammadex was an unexpected finding, and the underlying mechanism is unknown. However, all values were within the normal range, and we did not perform another test for fibrinolysis in our patients. Therefore, clinical relevance is limited for this finding. We expected that an increase in sugammadex concentration in the whole blood by 10 μg mL^− 1^ would result in an increase in the r time by 4.8 s and suggested that coagulation might be compromised when the blood concentration of sugammadex exceeded 500 μg mL^− 1^, which corresponded to an increase in the r value of > 10 min. This blood concentration could be expected after administration of a sugammadex dose >  32 mg kg^− 1^. Our findings are in accordance with the results of the recent clinical in vivo ROTEM study. By using INTEM® (thromboelastometry assay using egallic acid, phospholipid, and calcium), Carron and co-workers showed that each 100 mg increase in sugammadex dose increased the clotting time by approximately 5.2 s in morbidly obese patients [11]. In the in vitro ROTEM study, Dirkmann and colleagues showed an increase in the INTEM® and EXTEM® (thromboelastometric assay using tissue factor, phospholipid, and calcium chloride) clotting times and a decrease in the activity of the intrinsic pathway-associated factors (VIII, IX, XI, and XII) [12]. However, they suggested that the affinity of sugammadex for phospholipid binding might interfere with the results of various coagulation studies that use phospholipids. The mechanism responsible for the anticoagulant effects of sugammadex is not clear. De Calm and co-workers suggested that the anticoagulant effects were related to the inhibition and induction of the activity of the intrinsic pathway-associated factor (Xa) [2]. The changes in the TEG parameters observed in the present study were similar to those reported in a previous in vitro study that used oral direct factor Xa inhibitors and direct anti-thrombin inhibitors [4]. R time, k time, α angle, MRTG, and TMRTG were statistically different in factor Xa inhibitor or direct thrombin inhibitors when compared with the control. Pipilis and co-workers found that among 75 patients with atrial fibrillation, those with high plasma dabigatran concentrations exhibited an r time > 11 min and an aPTT > 65 s, with a correlation between the r time and aPTT [5]. In patients receiving warfarin, TEG and ROTEM showed an acceptable diagnostic value [13]; in addition, the PT-INR and TEG r times correlated with thrombin generation and were corrected after prothrombin complex administration in patients with intracranial bleeding who were treated with warfarin [14]. Therefore, TEG may offer a valuable tool for the assessment of the effectiveness of the management of perioperative bleeding in patients receiving warfarin or direct oral anticoagulants. Moreover, it may provide coagulation profiles for patients undergoing non-elective surgery who have recently received anticoagulant therapy.

Additionally, patients who are actively bleeding and/or have altered hemostasis are expected to be admitted to the ICU and may need point of care viscoelastic testing as well as other laboratory exams. Delayed weaning from mechanical ventilation would be a more appropriate choice until the establishment of adequate transfusion, the correction of acidosis and hypothermia, and other supportive treatments for coagulation abnormalities.

Our study has several limitations. First, it is an in vitro study; therefore, the extent to which the results can be translated into the clinical setting is limited. However, as sugammadex is not metabolized after IV injection, our results may be reasonably extrapolated to in vivo effects. Furthermore, in the clinical scenario, the r time is usually shortened, while the increase or decrease of the α angle and MA by mild hemodilution depends on whether native or citrated blood is used [15, 16]. Therefore, the expected changes related to the clinical dose of sugammadex may not be easily detected by using in vivo TEG in surgical patients. Postoperative hypercoagulability, due to a decrease in anti-thrombin after surgery, might reduce the magnitude of the changes in coagulation parameters induced by sugammadex [17]. In an observational study of cancer surgery and other minor surgeries, PT and aPTT were not altered with sugammadex use [18, 19]. Second, we used citrated blood for a practical reason: the TEG analysis of all samples could not be performed within 4 min of the collection of blood samples. The use of citrated blood in TEG analysis could yield different results from those measured in samples of native, non-citrated blood [20].

## Conclusions

Based on the finding of this in vitro study, a whole blood sugammadex concentration above 193 μg mL^− 1^ would enhance hypocoagulability on thromboelastography of patients without coagulation abnormality. Future clinical studies are needed to examine the effects of sugammadex on the coagulation profiles of patients with altered hemostasis and, importantly, to examine outcomes related to sugammadex exposure related to enhanced hypocoagulablilty.

## Abbreviations

ASA: American Society of Anesthesiologists
G: shear elastic modulus strength
k: coagulation time
Ly 30: lysis 30
MA: maximum amplitude
MRTG: Maximum rate thrombus generation
r: reaction time
TEG: Thromboelastography
TMRTG: Time to maximum rate thrombus generation
TTG: Total thrombus generation
α: angle
